# Supplementary material for: scDART: integrating unmatched scRNA-seq and scATAC-seq data and learning cross-modality relationship simultaneously
Source: Genome Biol. 2022 Jun 27;23:139. doi: 10.1186/s13059-022-02706-x (PMC9238247; doi:10.1186/s13059-022-02706-x)
Supplement: Supplementary file 1 — Additional file 1 Supplementary figures S1-S8. [file 13059_2022_2706_MOESM1_ESM.pdf]

Additional file 1: Supplementary Figures for "Integrating unmatched scRNA-seq and scATAC-seq data and learning cross-modality relationship simultaneously"

Supplementary Figures

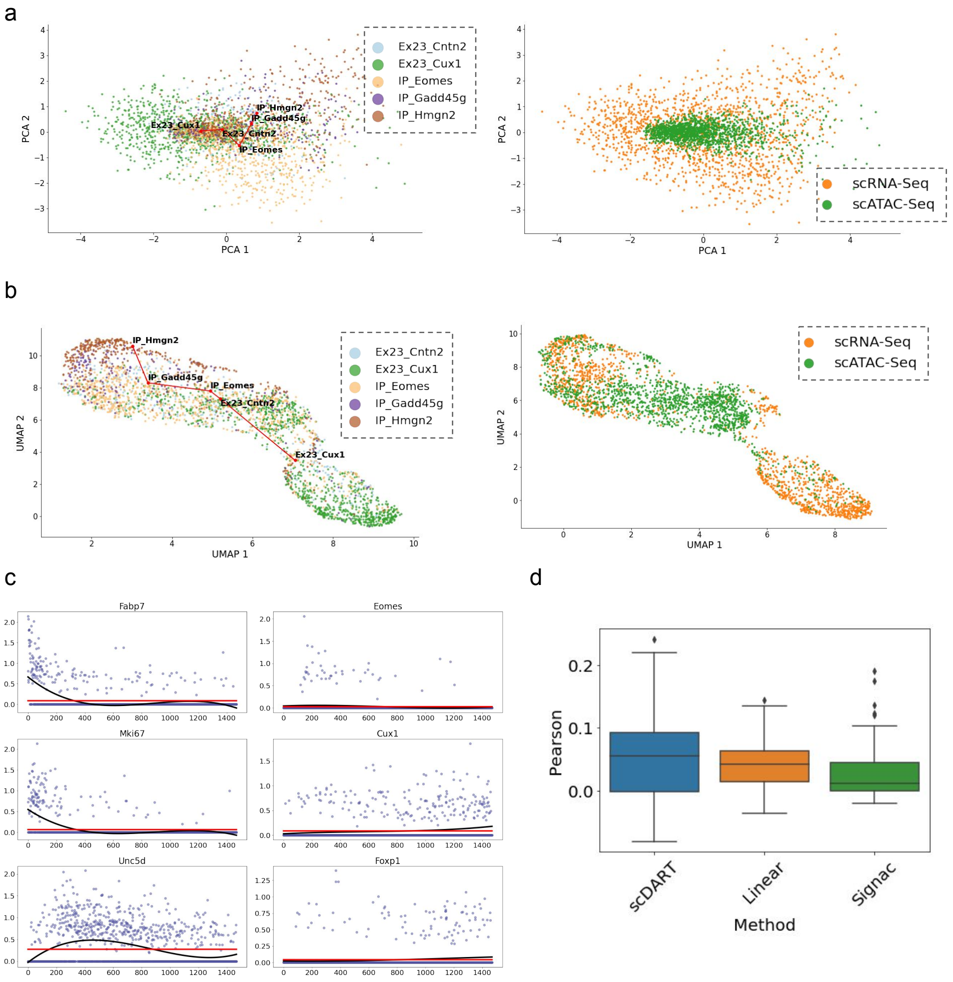

Figure S1: Additional test results of scDART and other baseline methods on the SNARE-seq mouse neonatal brain cortex dataset. **a** Latent embedding of integrated data obtained with `MMD-MA`. Left: cells colored with cell type annotations from the original paper and the inferred trajectory backbone is shown; Right: cells colored with batches (or modality). **b** Latent embedding of integrated data obtained with `scJoint`. Left: cells colored with cell type annotations from the original paper and the inferred trajectory backbone is shown; Right: cells colored with batches (or modality). **c** Expression levels of *Mki67*, *Fabp7*, *Unc5d*, *Cux1*, *Eomes*, and *Foxp1* along the pseudotime inferred from measured scRNA-seq data. The black and red lines correspond to the fitted statistical models under alternative and null hypothesis, respectively, when conducting likelihood ratio test. **d** Boxplot of Pearson correlation between pseudo-scRNA-seq and ground truth scRNA-seq inferred by scDART, the linear method (used in Seurat v3) and Signac.

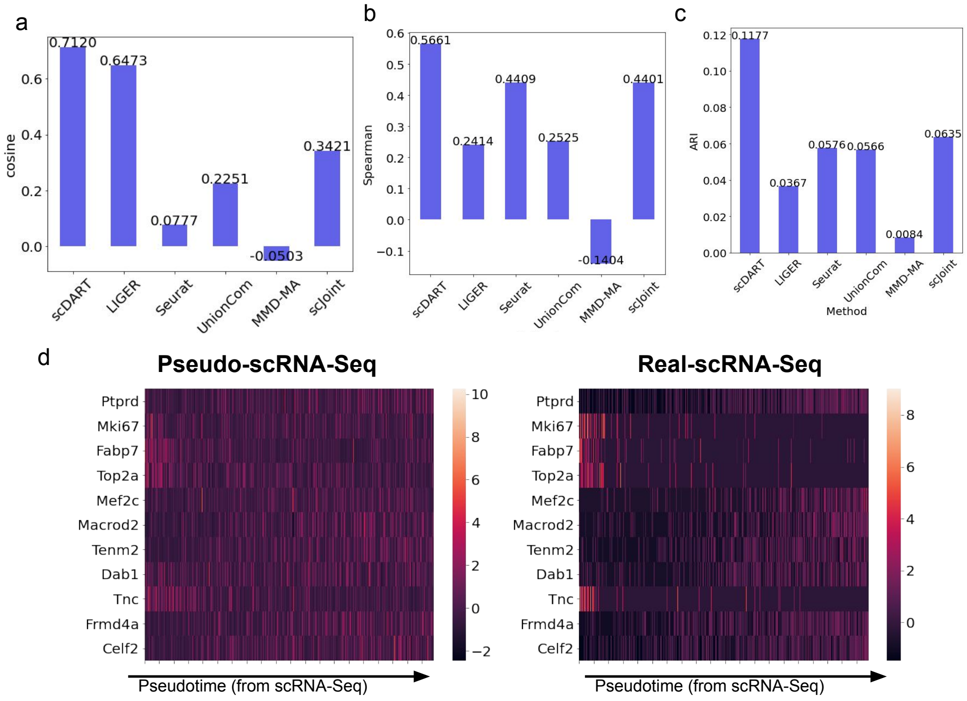

e

scDART

Gene	Spearman	Pearson
Ptprd	0.0479	0.0655
Mki67	0.1376	0.1846
Fabp7	0.1541	0.2412
Top2a	0.1236	0.2030
Mef2c	0.0973	0.0447
Macrod2	0.1320	0.1529
Tenm2	0.0932	0.0849
Dab1	0.0517	0.0624
Tnc	0.1783	0.2189
Frmd4a	0.1507	0.1371
Celf2	0.0178	0.0227

f

Linear

Gene	Spearman	Pearson
Ptprd	0.0591	0.0693
Mki67	0.0639	0.0426
Fabp7	0.1564	0.1346
Top2a	0.0354	0.0284
Mef2c	0.1436	0.1240
Macrod2	0.0588	0.0489
Tenm2	0.0629	0.0528
Dab1	0.0834	0.0823
Tnc	0.0874	0.0638
Frmd4a	0.0682	0.0710
Celf2	0.0701	0.0766

Figure S2: Additional testing results of scDART on mouse neonatal brain cortex dataset. **a** Cosine similarity score of scDART and baseline methods. **b** Pseudotime consistency score of scDART and baseline methods, where Spearman correlation is used. **c** Cluster consistency of scDART and baseline methods, where Adjusted Rand Index (ARI) is used to measure the consistency. **d** Pseudo-scRNA-seq (left) and real scRNA-seq (right) data of selected DE genes in cells ordered on the x-axis according to pseudotime inferred from real scRNA-seq data. Colors show the gene expression levels normalized into z-scores. **e** Correlation scores between pseudo-scRNA-seq and real scRNA-seq data of top DE genes. The pseudo-scRNA-seq data is obtained with `scDART`. **f** Correlation score between pseudo-scRNA-seq and real scRNA-seq data. The pseudo-scRNA-seq data is obtained with linear transformation on the scATAC-seq data with the input GAM.

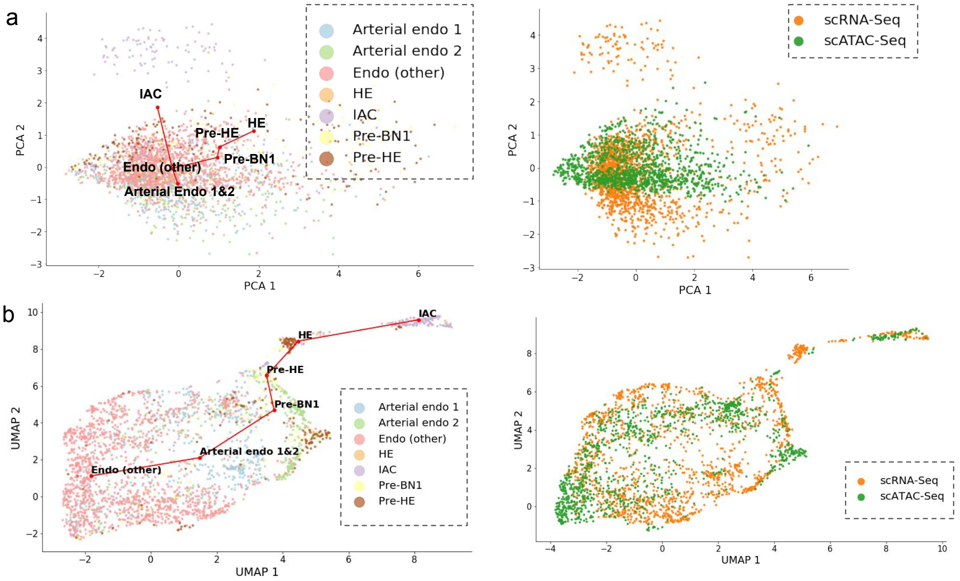

c

GO Term	Annotation	P-value
GO:0072091	regulation of stem cell proliferation	7.00E-05
GO:0010720	positive regulation of cell development	0.00035
GO:0030099	myeloid cell differentiation	0.00524

d

Motif	TF (sub-class)	Sequence logo	GO: Biological Process
MA0035.3_Gata1	GATA	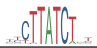	immune system process;cell differentiation
MA0482.1_Gata4	GATA	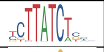	cell differentiation
MA0002.2_RUNX1	RUNX1	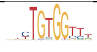	immune system process;cell differentiation
MA0087.1_Sox5	SOX	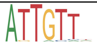	cell differentiation
MA0480.1_Foxo1	FOX	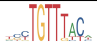	cell differentiation

Figure S3: Additional test results on the mouse endothelial dataset. **a** The latent embedding of `MMD-MA` on mouse endothelial dataset. Left: cells colored with cell type annotations from the original paper, and the red lines show the inferred trajectory backbone; Right: cells colored with batches (or modality). **b** The latent embedding of `scJoint` on mouse endothelial dataset. Left: cells colored with cell type annotations from the original paper, and the red lines show the inferred trajectory backbone; Right: cells colored with batches (or modality). **c** Top gene ontology terms of DE genes on mouse endothelial cell development dataset. **d** Selected differentially accessible motifs on mouse endothelial cell development dataset.

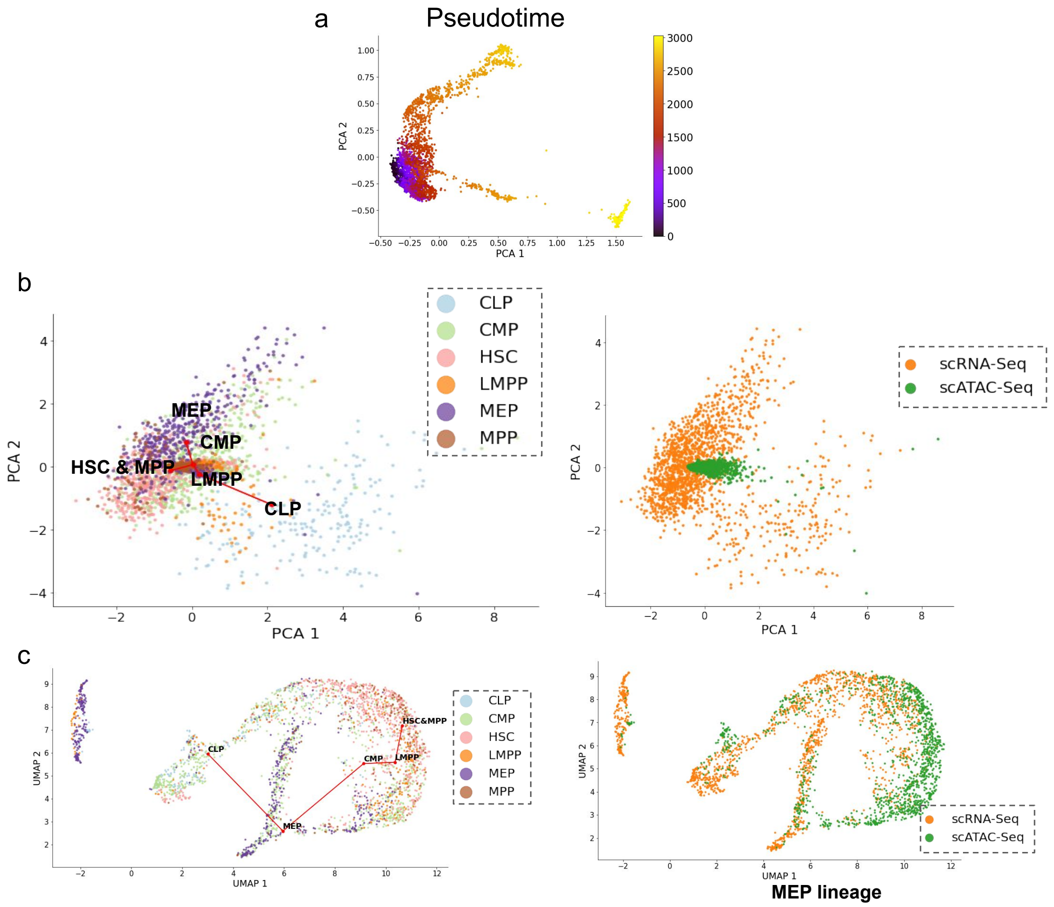

d

MEP lineage

GO Term	Annotation	P-value
GO:0051301	cell division	0.00014
GO:0002573	myeloid leukocyte differentiation	0.00743

CLP lineage

GO Term	Annotation	P-value
GO:0050853	B cell receptor signaling pathway	0.00452
GO:0045664	regulation of neuron differentiation	0.00605

e

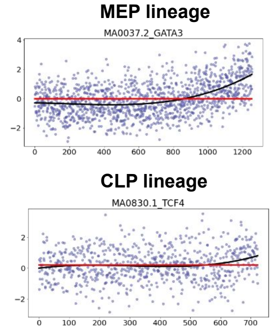

Figure S4: Additional test results on the human hematopoiesis dataset. **a** Latent embedding of `scDART` on human hematopoiesis dataset, where cells are colored with the inferred pseudotime. **b** The latent embedding of `MMD-MA` on human hematopoiesis dataset. Left: cells colored with cell type annotations from the original paper, and the red lines show the inferred trajectory backbone; Right: cells colored with batches (or modality). **c** The latent embedding of `scJoint` on human hematopoiesis dataset. Left: cells colored with cell type annotations from the original paper, and the red lines show the inferred trajectory backbone; Right: cells colored with batches (or modality). **d** The top gene ontology terms found by `TopGO` on human hematopoiesis dataset. **e** The deviation value (from ChromVAR) of differentially accessible motifs along MEP and CLP lineages. The black and red lines correspond to the fitted statistical models under alternative and null hypothesis, respectively, when conducting likelihood ratio test.

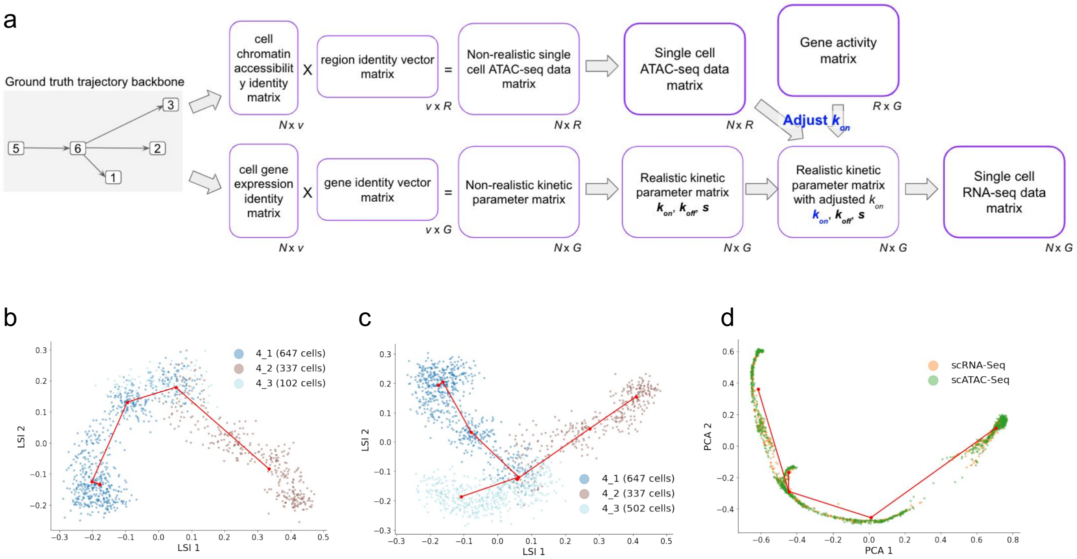

Figure S5: Additional test results on simulated datasets. **a** Illustration of the data simulation process. Given the ground truth trajectory and the gene activity matrix, the simulation procedure generates both scRNA-seq and scATAC-seq data. **b** The trajectory backbone learned from scATAC-seq where branch 4_3 has only 102 cells. **c** The trajectory backbone learned from scATAC-seq where branch 4_3 has 502 cells. **d** The trajectory backbone learned from `scDART` latent embedding where when branch 4_3 has 197 cells (95 from scRNA-seq and 102 from scATAC-seq). Cells are colored by data batches.

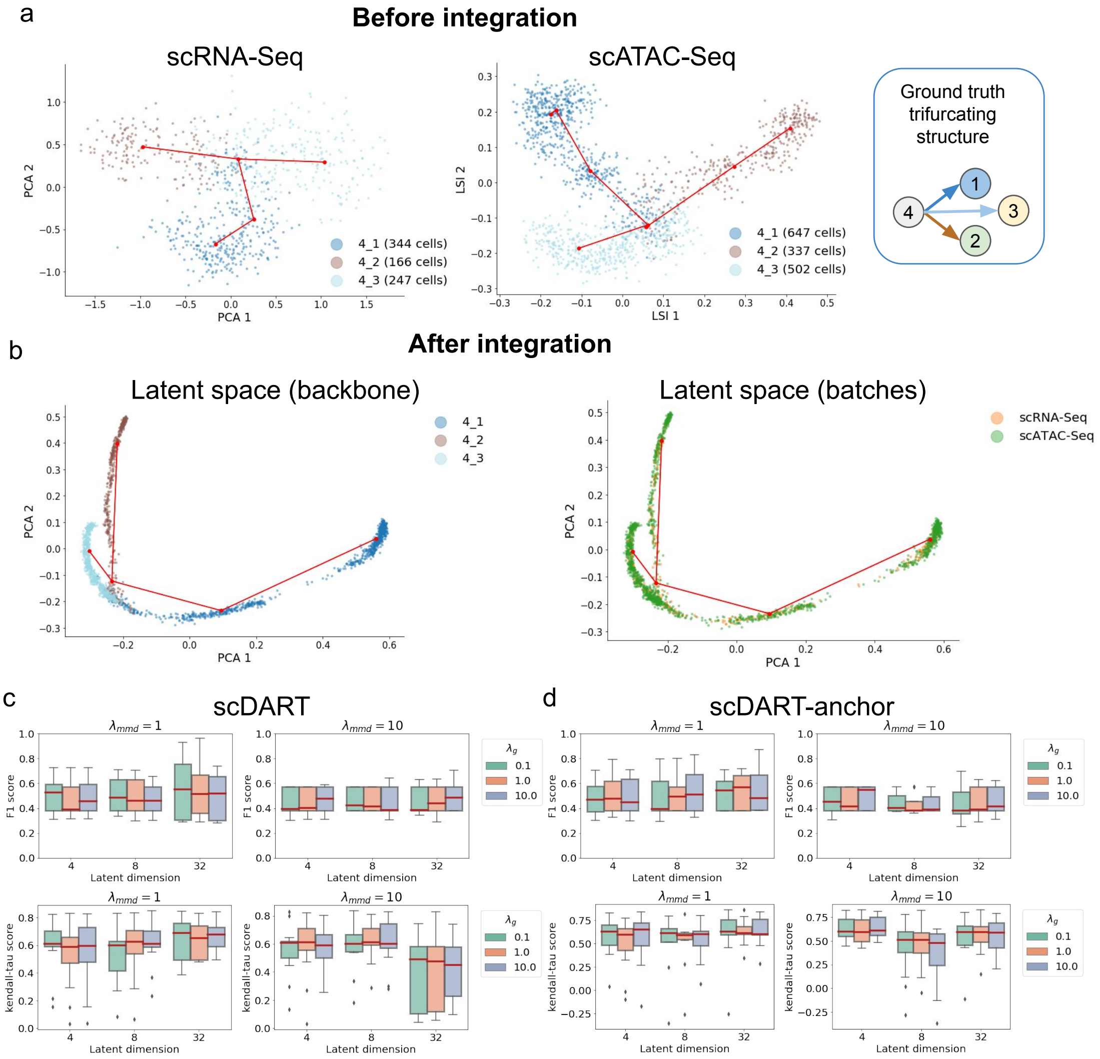

Figure S6: Additional test results on simulated datasets. **a** The PCA visualization of scRNA-seq batch which has only 757 cells (left) and scATAC-seq batch which has 1486 cells (right). The dataset has a trifurcating trajectory structure. **b** The latent embedding learned from `scDART` using the data from cell batches in subfigure a. Left: cells are colored by trajectory branches, and red line shows the inferred trajectory backbone. Right: cells are colored by data batches. **c-d** The boxplots of F1-score and Kendall-τ score under different hyper-parameter settings of (c) `scDART` and (d) `scDART-anchor`.

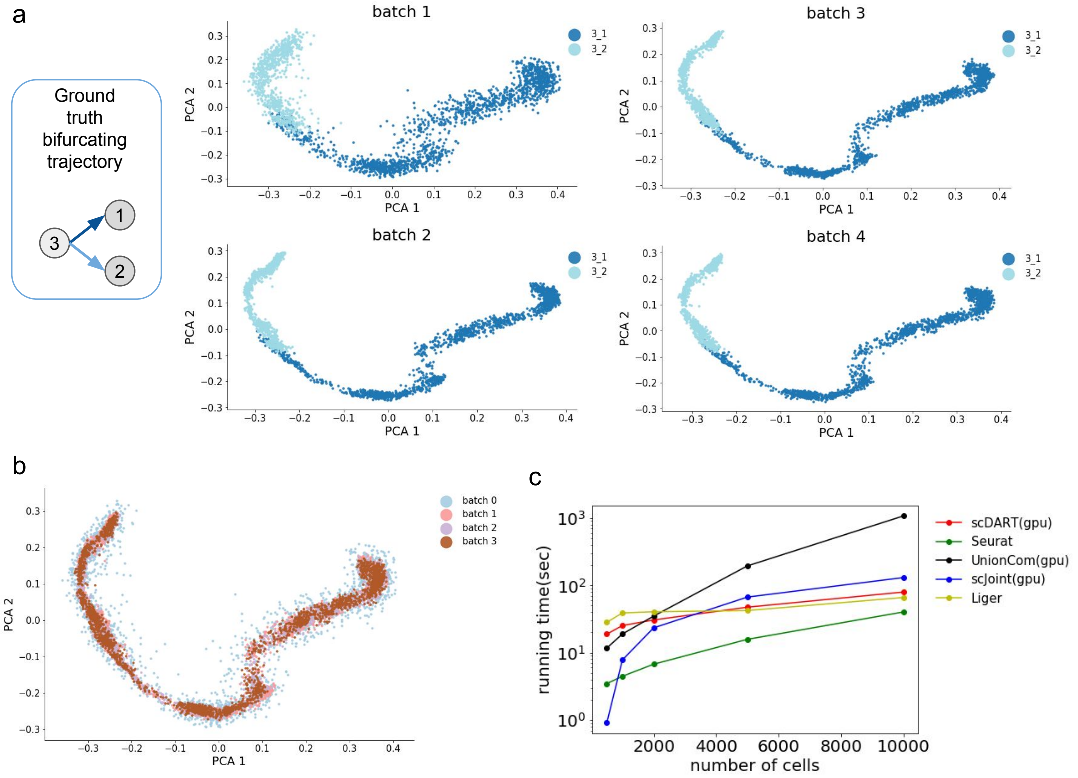

Figure S7: Additional test results. **a,b** The latent embedding of `scDART` on simulated dataset with four batches of cells, where batch 1 and 2 correspond to scRNA-seq, and batch 3 and 4 correspond to scATAC-seq. Cells are colored with (a) trajectory branches (b) data batches. **c** The running time of `scDART` compared with baseline methods.

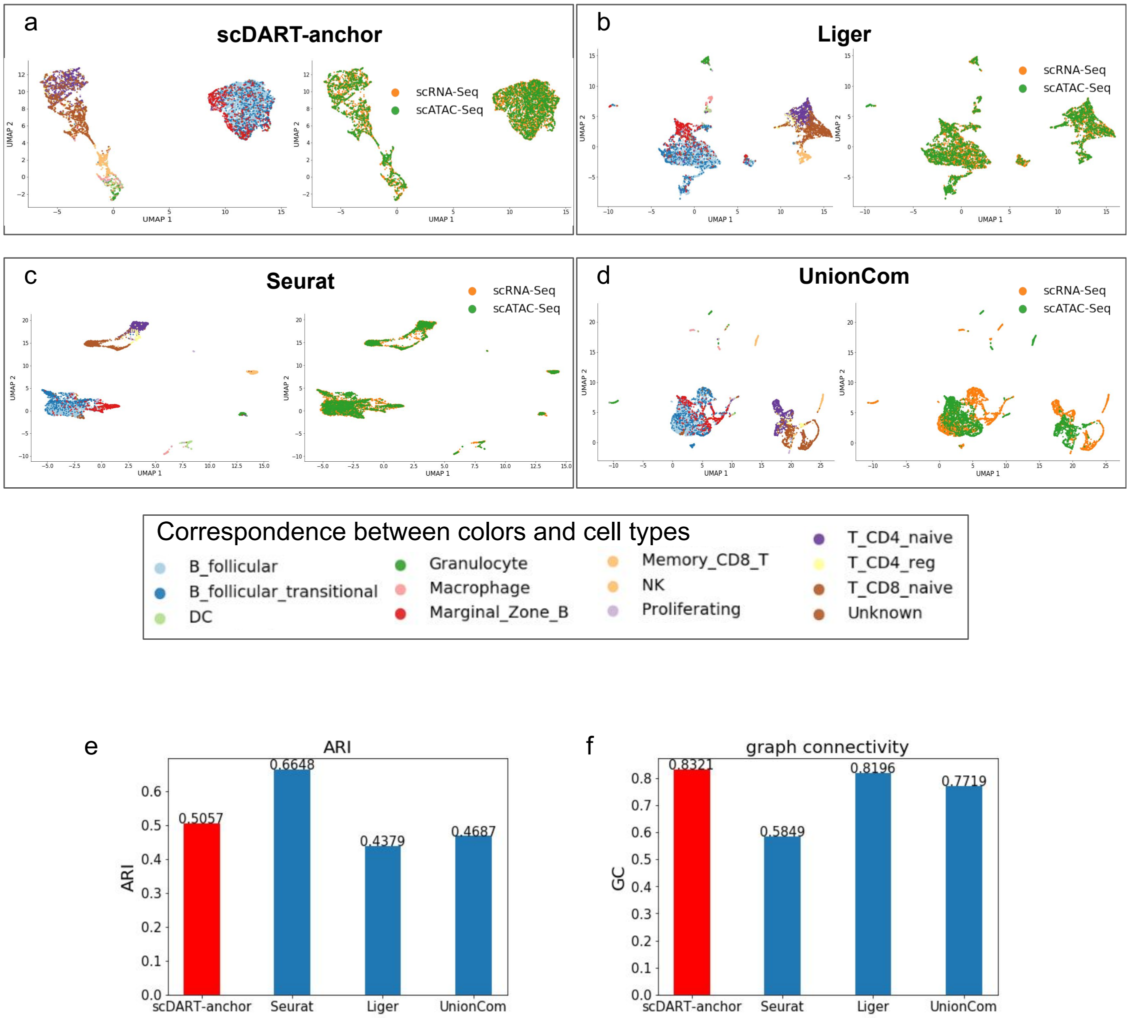

Figure S8: Test results on mouse spleen dataset. **a,b,c,d** The latent embedding of `scDART`, `Liger`, `Seurat`, and `UnionCom`, visualized using UMAP. For each method, the cells in the left figure are colored with ground truth cell types (legend in the box below all plots), and the cells in the right figure are colored with data modalities. **e** ARI scores of all methods. **f** Graph connectivity scores of all methods.
