# Supplementary material for: scDART: integrating unmatched scRNA-seq and scATAC-seq data and learning cross-modality relationship simultaneously
Source: Genome Biol. 2022 Jun 27;23:139. doi: 10.1186/s13059-022-02706-x (PMC9238247; doi:10.1186/s13059-022-02706-x)
Supplement: Supplementary file 4 — Additional file 4 Review history. [file 13059_2022_2706_MOESM4_ESM.docx]

Review History

**First round of review**

**Reviewer 1**

**Were you able to assess all statistics in the manuscript, including the appropriateness of statistical tests used?**

no

**Comments to author:**

The authors develop a method named scDART to perform a joint analysis of scRNA-seq and scATAC-seq measured on different cells. This method is also able to infer the cis-regulatory relationship. A huge amount of scRNA-seq and scATAC-seq data have been generated and these data require an efficient computational method to analyze. Thus, the method developed by authors is tackling an important problem. However, I have some concerns about the evaluation of the method.

1. A description of tuning parameters is needed. How many tuning parameters and how many fixed parameters does scDART have? How to choose parameters for scDART and what parameters are used for other methods? What is the dimension of latent space of scRART and other methods?

2. In Fig.2C, the performance of scDART is worse than Seurat when the neighborhood size is small (maybe 20 or 30). To detect minor populations, one cannot set the neighborhood size too big. For a dataset with 1469, a neighborhood with a size over 200 is not meaningful. Further evidence is needed to show the advantage of scDART compared to Seurat.

3. To evaluate the performance of embedding, the authors should compare the clustering aspect on SNARE-Seq data. Clustering should be performed on the latent space and each cell will obtain two labels, one based on scRNA-seq and the other based on scATAC-seq. The consistency of these two labels reflects the accuracy of embedding.

4. The pseudo-scRNA-seq count matrix should be evaluated. The SNARE-Seq data provide the real gene expression of each cell, so one can evaluate the performance of the pseudo-scRNA-seq count matrix by comparing it with the real expression data. The same comparison should be performed on the gene activity score from Seurat, which is similar to the pseudo-scRNA-seq count matrix. If the pseudo-scRNA-seq count matrix is more consistent with the real gene expression than the gene activity score defined in Seurat, then it would be a strong validation for scDART.

5. Comparison with other methods, like recently published method scJoint.

scJoint integrates atlas-scale single-cell RNA-seq and ATAC-seq data with transfer learning.

**Reviewer 2**

**Were you able to assess all statistics in the manuscript, including the appropriateness of statistical tests used?**

yes

**Comments to author:**

The paper proposes a neural network based method to integrate unmatched scRNA-seq and scATAC-seq data, with the main novelties being (1) the focus on trajectory data, (2) a module that learns a nonlinear gene activity function from scATAC-seq data. Given the multitude of recent methods for integrating unmatched single cell data from different modalities, there are aspects of the paper that need further clarification before evaluating its merits.

- I am not entirely convinced that preserving the distance in the original data space (PCA or LSI transformed) as done in the loss L_dist is the best thing to do. One can easily think of situations where noise in the data, batch effects (within one modality), obscure the true trajectory. Can scDART handle batch effects that exist in each modality?

- scDART applies a post-processing step to the latent space to obtain cleaner trajectories. How much does this step affect the trajectory comparison results on the real datasets? Can we apply the same post-processing step to the latent space of the baseline methods (Liger, Seurat, etc.) to obtain similar improvement?

- It is stated that scDART "also works for cells that form discrete clusters", but this claim is not substantiated by real data examples; the only simulated data under this setting appears to have a simplistic structure (Fig 5e). While I understand the method is designed mainly for trajectory data, the authors would need to provide more supporting evidence or further discussion.

- I appreciate that the paper presents three real data applications of scDART. However, all the examples are very similar in data structure and data size. It would make the case more convincing if more challenging variations in the data can be considered, for example, batch effects within each modality, imbalanced data sizes for scRNA-seq and scATAC-seq, a different modality (methylation or protein data). Could the authors also comment on the runtime and scalability of scDART?

- The paper compares the gene activity scores from scDART with those obtained from a linear method. Could the authors clarify if the comparison was performed against widely used methods, such as Signac and Cicero? The correlations from scDART still appear to be on the low side in Fig 2e.

- Seurat consistently failed to mix the two modalities in all the real datasets, which is somewhat surprising. As per standard practice, descriptions of how each dataset was pre-processed and the parameters used in each baseline method need to be given.

- Details of the neural network architecture (number of nodes in each layer), optimization details (batch size, epochs, step size of SGD), how each data was pre-processed for scDART need to be given.

**Authors Response**

**Point-by-point responses to the reviewers’ comments:**

Reviewer #1

The authors develop a method named scDART to perform a joint analysis of scRNA-seq and

scATAC-seq measured on different cells. This method is also able to infer the cis-regulatory

relationship. A huge amount of scRNA-seq and scATAC-seq data have been generated and

these data require an efficient computational method to analyze. Thus, the method developed

by authors is tackling an important problem. However, I have some concerns about the

evaluation of the method.

1. A description of tuning parameters is needed. How many tuning parameters and how many

fixed parameters does scDART have? How to choose parameters for scDART and what

parameters are used for other methods? What is the dimension of latent space of scDART and

other methods?

*1) Tuning parameters: there are 3 tuning parameters for scDART, including the number of*

*dimensions in the latent space d, the weights of the loss terms in the loss function, λg*

*and λmmd.*

*Fixed parameters: fixed parameters include the neural network architecture and the*

*number of neighbors used in the post-processing step. We include the parameter setting*

*of the neural network in Table 1 (page 28), and the number of neighbors is fixed to be 10*

*(specified in section Post-processing after training, page 18).*

*2) In subsection “Effects of hyper-parameters” (page 11-12) we discuss the effects of the*

*three tuning parameters, d, λg and λmmd, This section also has default values of each*

*parameter and guidance on when to increase or decrease a certain parameter. In this*

*section, we have also tested the robustness of scDART and scDART-anchor against the*

*changes in the tuning parameters.*

*We added a section Settings of scDART and baseline methods (page 22) to describe the*

*detailed settings of scDART (including the settings of tuning and fixed parameters) and*

*settings of the baseline methods.*

*3) The dimension of latent space of scDART and baseline methods are also included in the*

*section Settings of scDART and baseline methods on page 22.*

2. In Fig.2C, the performance of scDART is worse than Seurat when the neighborhood size is small (maybe 20 or 30). To detect minor populations, one cannot set the neighborhood size too big. For a dataset with 1469, a neighborhood with a size over 200 is not meaningful. Further evidence is needed to show the advantage of scDART compared to Seurat.

*We thank the reviewer for pointing this out which led us to perform further investigation. First, we looked into the definition of the neighborhood overlap score and found this evaluation metric has its limitations. According to existing literature, the neighborhood overlap score is defined as follows: given a neighborhood size k, a k-nearest neighbor graph on the latent embedding of cells from both scRNA-seq and scATAC-seq data is constructed. The neighborhood overlap score calculates the proportion of cells that have their matching cells in the other modality included within their neighborhoods. In this manner, the cell pairs considered are only the cells that fall into the same neighborhood. If two matching cells do not fall into the same neighborhood but have large differences between them, this is not penalized in the neighborhood overlap score. To complement the neighborhood overlap score, we used an additional measurement, which is the cosine similarity score to quantify the accuracy of different methods on matching the cell pairs between the scRNA-seq and the scATAC-seq modalities. The cosine similarity score calculates the similarity between the learned embedding of the cell in scRNA-seq data and its corresponding cell in scATAC-seq data, and it considers all the cells. The results of all methods are in the following figure (added to the manuscript as Figures S2a). Cosine similarity score of the latent embedding of different modalities:*

*scDART has overall the best performance out of all methods considering both the neighborhood overlap score and the cosine similarity score. The superior performance of scDART is also supported by other metrics including the pseudo-time consistency score (Fig. 2d, Fig. S2b). We have modified the manuscript (Results: page 6, top paragraph, Methods: page 21) to reflect the new results on cosine similarity.*

3. To evaluate the performance of embedding, the authors should compare the clustering aspect on SNARE-Seq data. Clustering should be performed on the latent space and each cell will obtain two labels, one based on scRNA-seq and the other based on scATAC-seq. The consistency of these two labels reflects the accuracy of embedding.

*According to the reviewer’s suggestion, we ran k-means clustering algorithm to cluster the latent embedding of scRNA-seq data and scATAC-seq data separately, and measure the clustering consistency by calculating the ARI (Adjusted Rand Index) score between the clustering results respectively from the scRNA-seq and scATAC-seq data. The number of clusters in the k-means algorithm is set to be 5, which is the number of ground truth cell types in the dataset. The resulting barplot is shown below (also added as Fig. S2c). scDART and scJoint have the best performance compared to other methods. However, one point to note is that the SNARE-Seq dataset does not have a strong clustering pattern (cells form continuous trajectory as shown by PCA), and the results of the clustering algorithm can be sensitive to small perturbations on the latent embedding.*

*We have also modified the text to reflect this additional result (Results: page 6; Methods: page 21-22).*

4. The pseudo-scRNA-seq count matrix should be evaluated. The SNARE-Seq data provide the real gene expression of each cell, so one can evaluate the performance of the pseudo-scRNA-seq count matrix by comparing it with the real expression data. The same comparison should be performed on the gene activity score from Seurat, which is similar to the pseudo-scRNA-seq count matrix. If the pseudo-scRNA-seq count matrix is more consistent with the real gene expression than the gene activity score defined in Seurat, then it would be a strong validation for scDART.

*The “linear method” that is compared with scDART in Fig. 2e and Fig. 5e refers to the procedure used in Seurat v3, where a region-gene relationship matrix obtained from the location of regions and genes on the genome was multiplied to the scATAC-seq matrix to obtain the pseudo-scRNA-seq.*

*Seurat v4 uses the procedure in Signac to calculate the gene activity score (the pseudo-scRNA-seq). Compared to the linear method, Signac directly calculates the overlap between regions and gene bodies on the genome from the fragment file and skips the peak calling process. The resulting pseudo-scRNA-seq is not affected by peak calling.*

*In Fig. 2e we compared the Pearson correlation between the pseudo-scRNA-seq and the ground-truth scRNA-seq data for the linear method (Seurat v3) and scDART. To also compare with Signac (Seurat v4), we calculate the Pearson correlation for top 50 differentially expressed genes along the trajectory and show the result of each method using a box in the boxplot below (also added as Fig. S1d). scDART infers pseudo-scRNA-Seq count matrix more accurately than the baseline methods according to these results. We also observe that the mean scores of Signac are lower than that of the linear method. This is due to that many genes in the given scRNA-seq data are not mapped to any chromatin fragments by Signac and the pseudo-scRNA-seq counts of these genes are thus 0s, which affected the overall correlation. These results suggest that, although avoiding errors introduced during peak calling can be an advantage of methods like Signac, one of their limitations is that they may not be able to predict the gene expression levels of certain genes.*

*We have modified the text to reflect these new results (page 6, bottom paragraph). On page 23 we include the details on how we ran Signac. Signac is not performed on the simulated data to test the accuracy of gene activity score prediction because it requires the BAM or segment files which are not available for simulated data.*

5. Comparison with other methods, like recently published method scJoint. scJoint integrates atlas-scale single-cell RNA-seq and ATAC-seq data with transfer learning.

*We run scJoint on all three real datasets: mouse neonatal brain cortex dataset (SNARE-seq), mouse endothelial cell developmental dataset, and human hematopoiesis dataset. We provided ground truth cell labels as input to scJoint. On each dataset, we ran scJoint using different parameter settings (center_weight={1, 20, 50, 100}, with_crossentropy={True, False}, and embedding_size={64, 32}) and chose the results which look best according to the visualizations. The results of scJoint on the three real datasets are shown below, which are also included as Fig. S1b, Fig. S3b and Fig. S4c. Corresponding discussions on these results are added to the manuscript (page 5, 7, and8). Details on how we ran scJoint are added to the manuscript (page 23).*

**Second round of review**

**Reviewer 1**

The authors have addressed all my concerns and I have no further comments.

**Reviewer 2**

The authors have addressed most of my comments, and I appreciate the effort of adding more case studies to evaluate the performance of scDART. I have two more follow-up questions.

- I notice that the visualization results of all the integration methods were done using PCA instead of other commonly used non-linear dimension reduction methods like UMAP and diffusion map. UMAP and diffusion map are more commonly used for trajectory visualization to better capture complex and continuous data structure, compared to PCA (e.g., see the benchmarking study by Luecken et al. (Nature methods, 2021), which examines the data integration of trajectory data via UMAP and diffusion map (Extended Fig 1-3)). I am wondering how the visualization comparisons of all the methods hold for the trajectory case studies when these two methods are used instead.

- Regarding the discrete population example using the mouse spleen dataset, the results need to be compared with the other baseline methods.

**Authors Response**

**Point-by-point responses to the reviewers’ comments:**

**Reviewer 2**

The authors have addressed most of my comments, and I appreciate the effort of adding more

case studies to evaluate the performance of scDART. I have two more follow-up questions.

- I notice that the visualization results of all the integration methods were done using PCA

instead of other commonly used non-linear dimension reduction methods like UMAP and

diffusion map. UMAP and diffusion map are more commonly used for trajectory

visualization to better capture complex and continuous data structure, compared to PCA

(e.g., see the benchmarking study by Luecken et al. (Nature methods, 2021), which

examines the data integration of trajectory data via UMAP and diffusion map (Extended Fig

1-3)). I am wondering how the visualization comparisons of all the methods hold for the

trajectory case studies when these two methods are used instead.

*We thank the reviewer for this question. First, we explain our choice of visualization method,*

*and then we show the UMAP and diffusion map visualizations of all methods on three real*

*datasets used in the manuscript.*

*Visualization methods like UMAP and TSNE aim to preserve the local structure (i.e. the*

*neighborhood of each cell) at the cost of losing global structure within the dataset (Kobak and*

*Berens 2019; Liu 2020). As a result, these methods may be able to show complex structure of*

*the data, but for the purpose of showing the trajectory structure of a dataset they are not ideal. A*

*commonly observed scenario is that when the cell density at a certain part of the trajectory is*

*low, the trajectory tends to be disconnected in UMAP, and instead, clusters are shown (Zhu et*

*al. 2020; Moon et al. 2019). This can also be observed in the Extended Data Fig. 1 of (Luecken*

*et al. 2021), where the integration results of the majority of methods are shown to form clusters.*

*On the other hand, PCA visualization, as a linear method, does not distort the structure of the*

*data like the nonlinear methods, and can better preserve the global trajectory pattern. However,*

*we agree that UMAP is an effective visualization method for data which discrete clusters. In our*

*manuscript, generally we have used PCA to visualize continuous datasets, and UMAP to*

*visualize discrete datasets.*

*Diffusion maps aim to preserve the diffusion distances between cells, so it can usually preserve*

*the trajectory structure. However, we noticed that in certain cases diffusion maps*

*“over-compress” cells with the same pseudotime to get thin trajectories, which make it hard to*

*observe different cell types on the trajectory. This can also be seen in some results in Extended*

*Data Figs. 2-3 of (Luecken et al. 2021).*

*It is recognized that every low-dimensional visualization method has its own shortcomings and*

*can only show partial information of the original high-dimensional data. We agree with the*

*reviewer that it is worth checking the comparison of different integration methods with different*

*visualization tools. In the following, we show the diffusion map and UMAP visualizations of our*

*method scDART and the baseline methods. The diffusion maps are generated with the “destiny”*

*package (Angerer et al. 2016). Discussions on the visualizations are in the figure caption of*

*each figure below.*

*1. UMAP and diffusion map visualizations on the mouse neonatal brain dataset*

*Figure 1. Diffusion map of scDART, Liger, Seurat, UnionCom, and scJoint on the mouse neonatal brain cortex dataset. The dataset has one linear trajectory. The expected trajectory is clearly shown in scDART results (top left plot), and the two batches are well mixed (top right plot). Liger almost shows a bifurcating trajectory. Seurat, UnionCom and scJoint do not merge the two batches. Seurat and UnionCom also do not show the expected trajectory.*

*Figure 2. UMAP of scDART, Liger, Seurat, UnionCom, and scJoint on mouse neonatal brain cortex dataset. The dataset has a linear trajectory. scDART is the method that shows the linear trajectory most clearly (observed from all plots in the left column) and scDART also shows the best batch mixing (from all plots in the right column).*

*2. UMAP and diffusion map visualizations on the mouse endothelial cell dataset*

*Figure 3. Diffusion map of scDART, Liger, Seurat, UnionCom, and scJoint on mouse endothelial cell dataset. The dataset has one linear trajectory starting from Endo (other) and ends at IAC. The IAC population is distant from the rest of the populations in all visualizations and HE has a very small number of cells that are hardly visible from these plots, so we focus on comparing the remaining part of the trajectory which is from Endo (other) to Pre-HE. scDART shows both the expected trajectory (top left plot) and good batch mixing (top right plot). UnionCom and scJoint do not mix the batches well from the diffusion map visualizations. In Seurat and Liger results (2nd and 3rd row, left plots) the Pre-HE population is not as concentrated as it is in the scDART result and are more mixed with other populations.*

*Figure 4. UMAP of scDART, Liger, Seurat, UnionCom, and scJoint on mouse endothelial cell dataset. This dataset has one linear trajectory starting from Endo (other) and ends at IAC. The IAC population is distant from the rest of the populations in all visualizations and HE has a very small number of cells that are hardly visible from these plots, so we focus on comparing the remaining part of the trajectory which is from Endo (other) to Pre-HE. scDART and Seurat best show the trajectory from Endo (other) to Pre-HE, but scDART shows a more even batch mixing than Seurat.*

*3. UMAP and diffusion map visualizations on the human hematopoiesis dataset*

*As is analyzed in the original manuscript, the human hematopoiesis dataset has two differentiating trajectories rooted at HSC which then change into MPP before differentiating into two fates, one goes to MEP and the other to CLP.*

*Figure 5. Diffusion map of scDART, Liger, Seurat, UnionCom, and scJoint on human hematopoiesis dataset. Starting from HSC and MPP, the cells terminate at two distinct cell fates: MEP and CLP. Out of all methods, scDART and Seurat are the only two methods that show the two distinct cell fates, and scDART shows a better batch mixing than Seurat.*

*Figure 6. UMAP of scDART, Liger, Seurat, UnionCom, and scJoint on human hematopoiesis dataset. Starting from HSC and MPP, the cells terminate at two distinct cell fates: MEP and CLP. UMAP separates certain populations into distant clusters which makes the trajectory not clear for any of the methods. Nevertheless, scDART best separates the populations and is the method that best matches the expected trajectory with a good batch mixing.*

- Regarding the discrete population example using the mouse spleen dataset, the results need to be compared with the other baseline methods.

*We run baseline methods including Liger, Seurat, UnionCom on the mouse spleen dataset, and*

*visualize the latent embedding using UMAP. The scDART results are the same as what we*

*showed in Supplementary Figure 7c of our last revision, and we include them here for*

*comparison with other methods. From the visualization, one can tell that UnionCom did not*

*merge the two batches well, but it is not straightforward to tell the performance difference*

*among scDART, Liger and Seurat. Therefore, we calculated quantitative measures to compare*

*the performance of these methods (Figure 8).*

*Figure 7. The UMAP visualization of the latent embedding of scDART, Liger, Seurat, and UnionCom*

*We quantitatively evaluate the latent embeddings output from the above methods using ARI (adjusted rand index) score and graph connectivity score as used in (Luecken et al. 2021). The ARI score calculates the separation of different cell types in the latent space (therefore evaluates how cell identity has been preserved during integration), whereas the graph connectivity score evaluates how well cell batches are mixed for each cell type. To calculate the ARI score, for each method, we ran the Leiden clustering algorithm on the latent embedding with different resolution parameters (from 0.1 to 1 with stepsize 0.5), and selected the one that leads to the best ARI for that method. The graph connectivity score was calculated by first constructing a k-nearest neighbor graph on the latent embedding, and measuring the connectivity of the sub-graph for each ground truth cell type (details in (Luecken et al. 2021)). The graph connectivity score is between 0 and 1, and higher score means better performance. Figure 8 shows the barplots of ARI score and graph connectivity score of different methods.*

*Figure 8. The barplots of ARI score (left) and graph connectivity score (right) of different methods*

*The barplot results show that scDART-anchor has the highest graph connectivity score and the second highest ARI score. Seurat, even though has the highest ARI score, has the lowest graph connectivity score. We observe that scDART-anchor performs comparably to, if not better than, the current state-of-the-art methods on this dataset. However, we opt to not emphasize the performance of scDART tools on discrete datasets, as we consider the strength of our methods and the focus of this work is the application on integrating cell trajectories of continuous datasets.*

*We have included Figure 7 and Figure 8 in this file as Supplementary Figure 8 of the revised manuscript. We have modified the manuscript accordingly to add the discussion on these results on page 12 and description of new metrics used on page 22 (changes highlighted in red).*

*References*

*Angerer, Philipp, Laleh Haghverdi, Maren Büttner, Fabian J. Theis, Carsten Marr, and Florian*

*Buettner. 2016. “Destiny: Diffusion Maps for Large-Scale Single-Cell Data in R.”*

*Bioinformatics 32 (8): 1241–43.*

*Kobak, Dmitry, and Philipp Berens. 2019. “The Art of Using T-SNE for Single-Cell*

*Transcriptomics.” Nature Communications 10 (1): 5416.*

*Liu, Zhenqiu. 2020. “Visualizing Single-Cell RNA-Seq Data with Semisupervised Principal*

*Component Analysis.” International Journal of Molecular Sciences 21 (16).*

*https://doi.org/10.3390/ijms21165797.*

*Luecken, Malte D., M. Büttner, K. Chaichoompu, A. Danese, M. Interlandi, M. F. Mueller, D. C.*

*Strobl, et al. 2021. “Benchmarking Atlas-Level Data Integration in Single-Cell Genomics.”*

*Nature Methods, December. https://doi.org/10.1038/s41592-021-01336-8.*

*Moon, Kevin R., David van Dijk, Zheng Wang, Scott Gigante, Daniel B. Burkhardt, William S.*

*Chen, Kristina Yim, et al. 2019. “Visualizing Structure and Transitions in High-Dimensional*

*Biological Data.” Nature Biotechnology 37 (12): 1482–92.*

*Zhu, Qin, Peng Gao, Joanna Tober, Laura Bennett, Changya Chen, Yasin Uzun, Yan Li, et al.*

*2020. “Developmental Trajectory of Prehematopoietic Stem Cell Formation from*

*Endothelium.” Blood 136 (7): 845–56.*
